# Ultrashort Echo Time Quantitative Susceptibility Source Separation in Musculoskeletal System: A Feasibility Study

**DOI:** 10.3390/jimaging12010028

**Published:** 2026-01-06

**Authors:** Sam Sedaghat, Jin Il Park, Eddie Fu, Annette von Drygalski, Yajun Ma, Eric Y. Chang, Jiang Du, Lorenzo Nardo, Hyungseok Jang

**Affiliations:** 1 Department of Diagnostic and Interventional Radiology, University Hospital Heidelberg, 69120 Heidelberg, Germany; 2 Department of Radiology, University of California, Davis, Sacramento, CA 95817, USA; 3 Department of Medicine, University of California, San Diego, San Diego, CA 92121, USA; 4 Department of Radiology, University of California, San Diego, San Diego, CA 92103, USA

**Keywords:** QSM, UTE, hemophilic arthropathy, source separation, MRI

## Abstract

This study aims to demonstrate the feasibility of ultrashort echo time (UTE)-based susceptibility source separation for musculoskeletal (MSK) imaging, enabling discrimination between diamagnetic and paramagnetic tissue components, with a particular focus on hemophilic arthropathy (HA). Three key techniques were integrated to achieve UTE-based susceptibility source separation: Iterative decomposition of water and fat with echo asymmetry and least-squares estimation for B0 field estimation, projection onto dipole fields for local field mapping, and χ-separation for quantitative susceptibility mapping (QSM) with source decomposition. A phantom containing varying concentrations of diamagnetic (CaCO_3_) and paramagnetic (Fe_3_O_4_) materials was used to validate the method. In addition, in vivo UTE-QSM scans of the knees and ankles were performed on five HA patients using a 3T clinical MRI scanner. In the phantom, conventional QSM underestimated susceptibility values due to the mixed-source cancelling the effect. In contrast, source-separated maps provided distinct diamagnetic and paramagnetic susceptibility values that correlated strongly with CaCO_3_ and Fe_3_O_4_ concentrations (r = −0.99 and 0.95, *p* < 0.05). In vivo, paramagnetic maps enabled improved visualization of hemosiderin deposits in joints of HA patients, which were poorly visualized or obscured in conventional QSM due to susceptibility cancellation by surrounding diamagnetic tissues such as bone. This study demonstrates, for the first time, the feasibility of UTE-based quantitative susceptibility source separation for MSK applications. The approach enhances the detection of paramagnetic substances like hemosiderin in HA and offers potential for improved assessment of bone and joint tissue composition.

## 1. Introduction

Magnetic susceptibility is one of the fundamental physical properties of matter, representing its response to an external magnetic field. Paramagnetic and ferromagnetic substances generate local dipole fields in the same direction as the applied magnetic field, whereas diamagnetic substances produce local dipole fields in the opposite direction. Examples of paramagnetic and ferromagnetic materials include iron, oxygen, magnesium, and gadolinium, while diamagnetic materials include calcium, silicon, carbon, and water. Magnetic susceptibility is dimensionless and has no unit; however, parts per million (ppm) or parts per billion (ppb) are commonly used to express its relative magnitude for practical purposes.

In an MRI system, tissues placed within the B0 field exhibit the same magnetic susceptibility effects. These effects can adversely impact image quality due to increased field inhomogeneity, particularly when tissues with strong susceptibility differences are present. This can lead to spatial distortion, phase errors, altered signal intensity, and blooming artifacts. However, quantifying susceptibility can reveal the biochemical composition of tissues in the human body [1,2,3,4,5,6]. Quantitative susceptibility mapping (QSM) has recently seen significant advancements, especially in neuroimaging, driven by innovations in signal processing and machine learning [7,8,9].

QSM serves as a powerful tool for probing tissue composition and identifying pathological changes. Alterations in susceptibility often reflect underlying shifts in chemical content—for instance, iron accumulation tends to increase susceptibility and has been implicated in various disease processes. In the liver, excessive iron deposition is a known hallmark of several liver disorders [10]. Similarly, abnormal iron buildup in the brain has been associated with neurodegenerative diseases such as multiple sclerosis and Alzheimer’s disease [11,12,13]. Susceptibility can also rise locally due to the presence of hemosiderin or microhemorrhages. On the other hand, a reduction in susceptibility may indicate the presence of calcium deposits, which can serve as important diagnostic markers in certain cancers [14].

The application of QSM in the musculoskeletal (MSK) system has also been explored [15,16,17]. Unfortunately, due to the short spin-spin relaxation time (T2) of many essential MSK tissues such as tendons, ligaments, meniscus, and bone, a conventional QSM based on gradient echo imaging only provides limited information in the long T2 tissues such as articular cartilage and muscle. Ultrashort echo time (UTE) based QSM (UTE-QSM) with a remarkably shortened TE can improve the sensitivity to the whole knee joint tissues, including both short and long T2 tissues [18,19,20]. In a recent study, the efficacy of UTE-QSM to detect highly accumulated hemosiderin (i.e., iron) in hemophilic arthropathy (HA) has been demonstrated [20].

More recently, novel approaches to separate susceptibility sources into diamagnetic and paramagnetic substances were proposed [21,22], which have been applied to imaging the brain to separate diamagnetic myelin from paramagnetic iron. However, the application of this technique to MSK imaging has not been explored yet. In this study, we demonstrate the feasibility of UTE-based quantitative susceptibility source separation targeting MSK tissues.

## 2. Materials and Methods

### 2.1. Phantom Design

A phantom of a mixture of diamagnetic and paramagnetic substances was prepared to validate the UTE-based quantitative source separation. The phantom comprised three 10 mL syringes with varying concentrations of calcium carbonate (CaCO_3_, Signa-Aldrich, Saint Louis, MO, USA). The concentrations of calcium carbonate were 522, 347, and 174 mg/mL, respectively. The concentrations of iron oxide (Fe_3_O_4_, Thermal Fisher Scientific, Ward Hill, MA, USA) were 12, 8, and 4 mg/mL, respectively. The mixed powders were immersed in 1% agarose gel to solidify completely.

### 2.2. In Vivo Subjects

In an in vivo experiment, five hemophilia patients with HA were recruited and underwent both knee and ankle MRIs.

MRI

**Figure 1:**
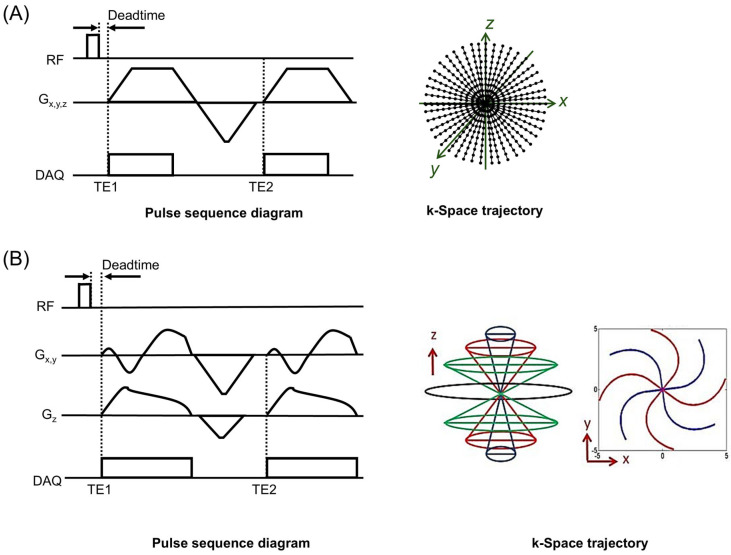
UTE pulse sequences. (**A**) Projection radial UTE and (**B**) cones UTE. Dual echo acquisition was utilized to acquire two images at TE1 and TE2. To obtain more echoes near ultrashort TE, image acquisition was repeated with delayed TE1 and TE2. (DAQ: data acquisiton).

MRI was performed in two 3T clinical MRI scanners: MAGNETOM Prisma (Siemens Healthineers, Erlangen, Germany) and MR750 (GE Healthcare, Chicago, IL, USA). Figure 1A,B show the pulse sequence diagram of the 3D projection radial UTE sequence implemented in 3T Siemens Prisma (used for the phantom experiment) and the 3D cones UTE sequence implemented in 3T GE MR750 (used for the in vivo experiment).

The imaging parameters were as follows: (1) Phantom experiment: A receive-only 20-channel head coil, flip angle = 10°, TR = 10 ms, TE = 0.05, 0.2, 0.4, 0.8, 2.2, 3.3, 4.4, and 5.5 ms (four dual echo scans), field of view = 200 × 200 × 192 mm^3^, matrix size = 200 × 200 × 96, and readout bandwidth = 80.6 kHz; (2) In vivo knee imaging: 8-channel transmit/receive knee coil, flip angle = 10°, TR = 10 ms, TE = 0.032, 0.2, 0.6, 2.7, 3.7, 4.7 ms (three dual echo scans), field of view = 150 × 150 × 86.4 mm^3^, matrix size = 220 × 220 × 96, and readout bandwidth = 250 kHz; (3) In vivo ankle imaging: 8-channel receive-only ankle coil and other parameters matched with the knee imaging.

All images were reconstructed into a complex form, using homemade software in MATLAB R2024b (Mathworks, Natick, MA, USA).

### 2.3. UTE-QSM with Source Separation

**Figure 2:**
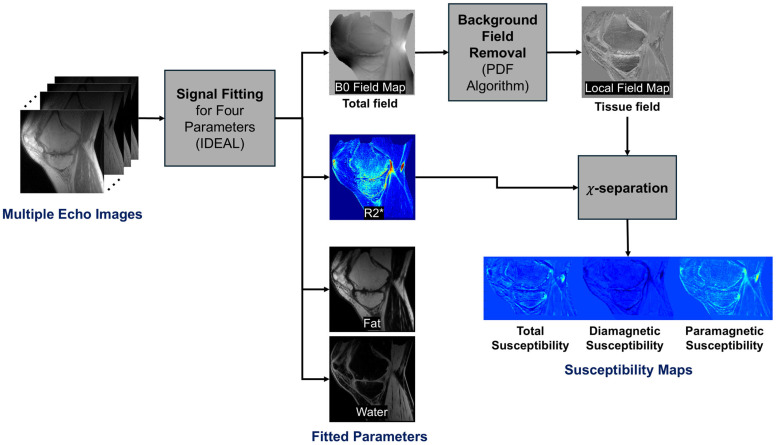
Framework of UTE quantitative susceptibility source separation. IDEAL-based fat and water signal fitting was performed to take into account the fat chemical shift in B0 field map estimation. The calculated B0 field map was processed with a PDF algorithm to yield a local field map. Subsequently, the local field map and R2* map were processed with *χ*-separation to yield total, diamagnetic, and paramagnetic susceptibility maps.

For UTE-based susceptibility source separation, three techniques were utilized and combined, including iterative decomposition of water and fat with echo asymmetry and least-squares estimation (IDEAL) to estimate a B0 field map [23], projection onto dipole field (PDF) to acquire a local field map [24], and *χ*-separation to achieve susceptibility source separation [22]. Figure 2 describes the workflow of UTE-QSM with source separation.

In contrast to common brain QSM, MSK QSM requires consideration of the fat signal. Fat protons exhibit a chemical shift of approximately −440 Hz relative to water protons at 3T, corresponding to a lower resonance frequency. This frequency shift can introduce significant errors in QSM, which relies on the phase evolution of off-resonance signals to estimate magnetic susceptibility. To address this issue, we employed IDEAL [23,25] utilizing a multi-peak fat spectral model consisting of six theoretical peaks with pre-calibrated relative amplitudes and frequency shifts. Furthermore, to accommodate the rapid signal decay typical of short-T2 tissues, the IDEAL algorithm jointly estimated the effective spin-spin relaxation time (T2*) of the water component, preventing phase errors associated with unmodeled signal decay.

IDEAL generates a B0 field map by fitting signals obtained at multiple echo times, incorporating both T2 decay and the fat chemical shift. The acquired complex signal was modeled according to this approach in gradient echo imaging at an echo time of *t*.

(1) $$S\left(t\right)=(S_{W}e^{-\frac{t}{T2*}}+S_{F}\sum_{k=1}^{N}\alpha_{k}e^{i2\pi f_{k}t})e^{i\;(\phi_{0}+2\pi f_{B0}t)},$$

In this model, *S_W_* and *S_F_* represent the magnitudes of the water and fat signals at zero TE, while *T*2* denotes the effective transverse relaxation time of the water signal. The parameters *α_k_* and *f_k_* correspond to the relative amplitudes and chemical shift frequencies of the multiple spectral peaks in fat (with *N* = 6 dominant peaks). The terms *ϕ*_0_ and *f*_*B*0_ represent the initial phase offset and the off-resonance frequency caused by B0 field inhomogeneities, respectively.

The resulting B0 field map was then processed using the PDF method to eliminate background field contributions and isolate the local field perturbations induced by tissue [24]. Then, the resultant local field map (i.e., tissue-generated field map) and R2* map (i.e., 1/T2*) are input to the susceptibility source separation pipeline implemented using *χ*-separation toolbox v1.1.3 (https://github.com/SNU-LIST/chi-separation, accessed on 18 October 2024) [22].

## 3. Results

### 3.1. Baseline Data

MRI scans of the knee and ankle joints were performed on five healthy male volunteers (age: 49.8 ± 15.2 years). All participants provided written informed consent before the imaging procedures.

### 3.2. Phantom Experiment

**Figure 3:**
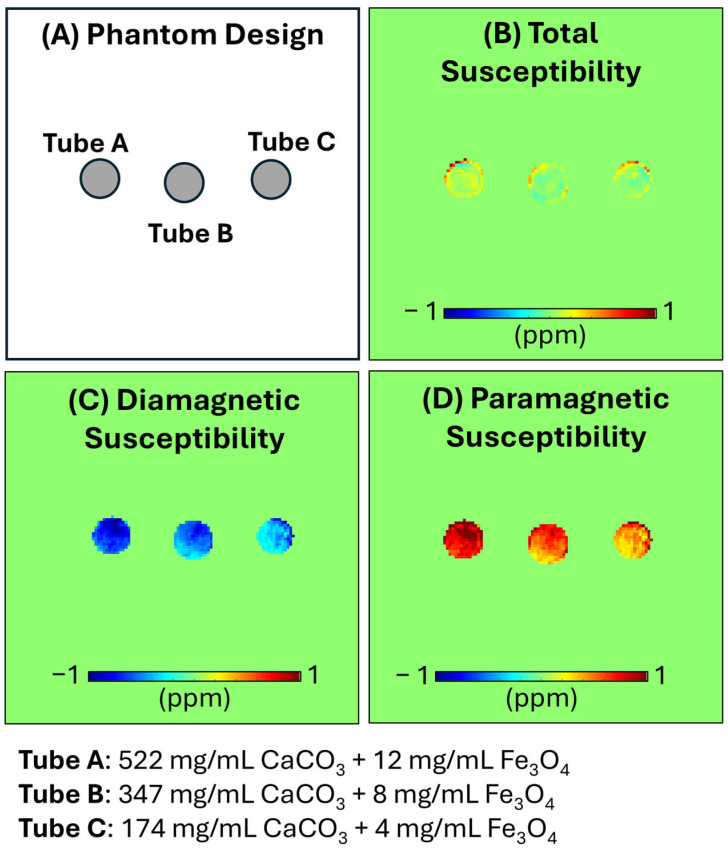
Phantom experiment. Three tubes were prepared by mixing diamagnetic calcium carbonate (CaCO_3_) and iron oxide (Fe_3_O_4_) in three different concentrations (CaCO_3_ of 522, 347,174 mg/mL and Fe_3_O_4_ of 12, 8, 4 mg/mL) (**A**). The estimated total susceptibility map cannot differentiate the three tubes because of the cancellation of the diamagnetic and paramagnetic sources (**B**). In contrast, the diamagnetic and paramagnetic susceptibility maps (**C**,**D**) clearly detect the CaCO_3_ and Fe_3_O_4_ with different concentrations, showing significant linear correlations (r = −0.99 and 0.95, *p* < 0.05).

Since the tubes contain mixed paramagnetic and diamagnetic sources (Figure 3A), the estimated total susceptibility was underestimated (Figure 3B), where the estimated susceptibility values were 0.153 ± 0.236, 0.040 ± 0.098, and 0.074 ± 0.138 ppm for the Tubes A, B, and C. The quantitative susceptibility source separation clearly detected the individual susceptibility sources. The estimated diamagnetic susceptibility values were −0.704 ± 0.149, −0.521 ± 0.145, and −0.410 ± 0.188 ppm for the three tubes (Figure 3C). The estimated paramagnetic susceptibility values were 0.857 ± 0.236, 0.560 ± 0.179, and 0.484 ± 0.264 ppm for the three tubes (Figure 3D). The separated paramagnetic and diamagnetic susceptibilities showed significant linear correlations with the CaCO_3_ and Fe_3_O_4_ concentrations, respectively (r = −0.99 and 0.95, *p* < 0.05).

### 3.3. In Vivo Experiment

In all five hemophilia patients, UTE-QSM with source separation provided reasonable separation of tissues with positive and negative susceptibilities.

**Figure 4:**
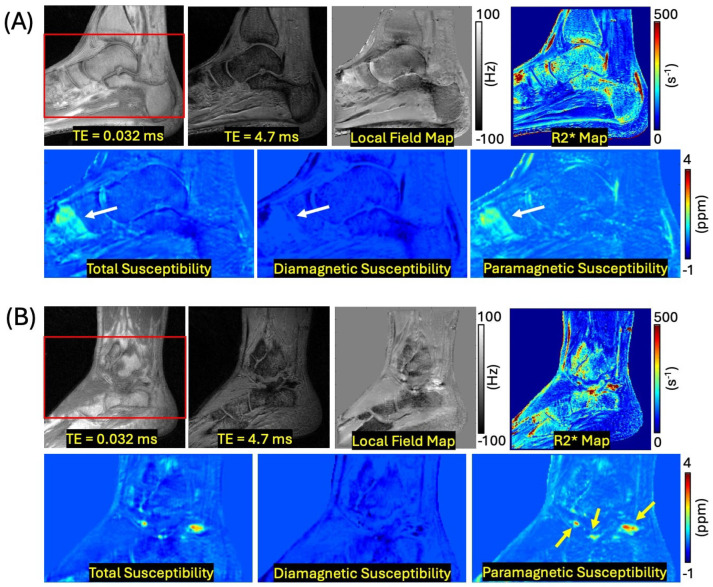
Ankle imaging with (**A**) a 52-year-old HA patient and (**B**) a 41-year-old HA patient. The bottom row shows zoomed-in susceptibility maps from the region indicated by the red box for each subject. The separated paramagnetic susceptibility map distinctly identifies tissues with high positive susceptibility; these tissues are intentionally suppressed and therefore absent on the diamagnetic susceptibility map, which selectively displays negative susceptibility sources (white arrows). In (**B**), the separated paramagnetic susceptibility map reveals hemosiderin deposition that is underestimated in conventional total susceptibility mapping due to susceptibility cancellation (yellow arrows).

Figure 4 shows the result of ankle imaging from two representative hemophilia patients. After susceptibility source separation, the paramagnetic susceptibility map clearly detects the tissues with positive susceptibility, which is obscured in the diamagnetic susceptibility map (white arrows in Figure 4A). In another HA patient, the separated paramagnetic susceptibility map clearly reveals the hemosiderin, which is underestimated in the conventional total susceptibility mapping (yellow arrows in Figure 4B).

**Figure 5:**
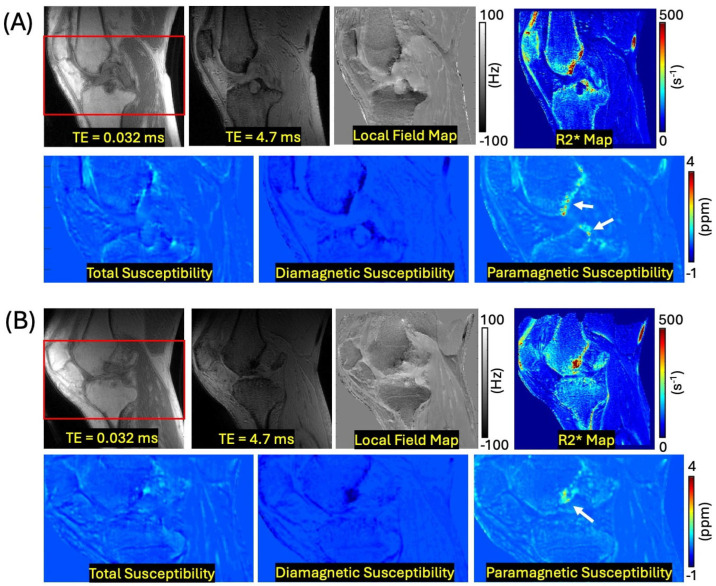
Knee imaging with (**A**) a 76-year-old HA patient and (**B**) a 51-year-old HA patient. The bottom row shows zoomed-in susceptibility maps from the region indicated by the red box for each subject. The separated paramagnetic susceptibility maps reveal the accumulated hemosiderin, which is not visible on the total susceptibility maps from conventional QSM presumably due to the cancellation caused by diamagnetic bone (white arrows).

Figure 5 shows the result of knee imaging from two representative hemophilia patients. The separated paramagnetic susceptibility maps detect the accumulated hemosiderin, which is not detected in the conventional QSM, presumably due to the cancellation caused by diamagnetic bone (white arrows in Figure 5).

## 4. Discussion

In this study, we demonstrated for the first time the feasibility of a UTE-based quantitative susceptibility source separation approach specifically targeting the MSK system. This method holds promise for providing a more accurate assessment of the chemical composition of short T2 tissues within MSK structures. We applied this approach to HA, a condition characterized by the accumulation of hemosiderin in joints, which contributes to degenerative changes in joint tissues. Since most joint tissues are diamagnetic except for adipose tissue, susceptibility source separation can enhance the accuracy of iron quantification by mitigating susceptibility cancellation effects. Another potential application of the proposed approach is UTE-QSM of bone, which significantly correlates with bone mineral density [18]. Due to the paramagnetic fat signal from bone marrow, the susceptibility values estimated in bone (diamagnetic) can be underestimated. The proposed UTE quantitative susceptibility source separation can be a promising method to address this issue.

In HA, sensitive and reproducible metrics are essential for evaluating both individual therapeutic response and overall treatment efficacy. Physical examination, even when performed by experienced clinicians, demonstrates limited reliability. Conventional radiography is restricted to detecting late-stage osseous abnormalities and lacks sensitivity to soft-tissue pathology, including synovitis and cartilage degeneration [26,27,28,29]. Although CT is highly sensitive to bony changes, it cannot adequately assess synovitis or cartilage degeneration [29]. Ultrasound offers advantages such as low cost, short acquisition time, and reliable detection of synovitis; however, its utility is constrained by limited acoustic windows and penetration, preventing comprehensive evaluation of intra-articular structures, as well as poor sensitivity to hemosiderin [30,31]. MRI remains the only validated gold standard for accurate joint assessment in HA [32,33,34]. While MRI provides superior soft-tissue contrast and high sensitivity to iron deposition and cartilage structural changes, it continues to face notable limitations, including imprecise semi-quantitative assessment of hemosiderin and limited ability to detect early iron accumulation and early degeneration in cartilage and subchondral bone [35]. The proposed UTE susceptibility source separation technique may provide an important clinical tool for HA diagnosis by enabling sensitive quantification of hemosiderin and concurrent assessment of subchondral bone changes.

In this setting, a quantitative imaging biomarker that is sensitive to iron deposition and less affected by surrounding bone is particularly valuable. The proposed UTE-based susceptibility source separation approach directly targets this unmet need by mitigating susceptibility cancellation and improving visualization of hemosiderin within affected joints. This capability may support earlier detection of joint involvement and more objective monitoring of disease progression and therapeutic response in hemophilic arthropathy.

A primary assumption of the *χ*-separation algorithm is the linear proportionality between R2 and R2*, which allows for the estimation of the reversible relaxation component R2’. Although the experimental results with the CaCO_3_ and Fe_3_O_4_ and the in vivo experiment showed promising results, we did not directly prove that this assumption is valid for hemosiderin with short T2 decay. While this assumption is standard in susceptibility source separation [22], its validity for hemosiderin, which exhibits extremely rapid T2 decay, has not been directly confirmed through traditional spin-echo sequences due to their inherent echo time limitations. However, theoretical frameworks for the static dephasing regime suggest that in the presence of large paramagnetic clusters like hemosiderin, both R2 and R2* should scale linearly with the iron volume fraction and its magnetic moment [36]. This linear relationship between relaxation rates and iron concentration has been empirically observed in various iron-overload studies [37,38,39], supporting the use of R2*-based models as a proxy for iron distribution. Despite these theoretical supports, the specific proportionality constant for hemosiderin may differ from that of ferritin or other iron-containing molecules, potentially introducing quantitative bias in the susceptibility maps. If the R2-R2* relationship is not consistent across different tissue environments, the algorithm might misestimate the susceptibility sources. Future validation using specialized UTE-spin-echo sequences is warranted to further refine these estimations in vivo. Unfortunately, measurement of R2 in hemosiderin is extremely challenging due to the long TE required for the spin echo sequence. One potential technique to measure T2 for the short T2 hemosiderin is the ultrashort echo time double echo steady state (UTE-DESS) technique [40].

Despite these challenges, the consistent separation observed in both phantom and in vivo experiments suggests that the R2*-based approximation is sufficiently robust for feasibility-level assessment in the current clinical setting. Future technical refinements and validation with specialized UTE spin-echo–based methods will be essential to improve quantitative accuracy and further establish clinical reliability.

A potential limitation of this study is the use of different UTE implementation strategies between the phantom (3D radial projection UTE sequence in Siemens Prisma) and in vivo (3D cones UTE sequence in GE MR750) experiments. Specifically, these sequences utilized different k-space sampling trajectories and readout bandwidths (80.6 kHz vs. 250 kHz). However, our previous investigation into UTE-QSM consistency demonstrated that susceptibility quantification remains highly robust across various encoding schemes, including radial and spiral cones [19], where we observed minimal differences in the estimated susceptibility values. Furthermore, varying the stretching factors of the cone spiral arms—which effectively alters the readout duration and gradient strength—resulted in negligible variations in estimated susceptibility values. These findings suggest that the fundamental magnetic properties captured by UTE-QSM are largely independent of the specific k-space trajectory and bandwidth settings used in this study, supporting the validity of comparing our phantom and in vivo results.

In cases of extremely high hemosiderin concentration, even UTE imaging may fail to detect signal from affected voxels. While UTE imaging significantly shortens the achievable echo time, extremely high concentrations of hemosiderin can still lead to signal voids, rendering R2* mapping and QSM estimation challenging in those specific regions. While QSM reconstruction can still be performed in these signal void regions using phase information from surrounding tissues, R2* mapping cannot provide data in such areas, as it relies on direct signal detection from the targeted tissue. This represents a fundamental limitation of the technique that cannot be overcome without further shortening the TE. In our current in vivo cohort (e.g., Figure 4 and Figure 5), we did not observe such extreme signal loss, and the susceptibility measurements remained stable across the visualized tissues. This absence of signal void is likely due to the relatively lower iron concentrations in these patients compared to those reported in ex vivo HA tissue studies [6], which demonstrated that localized regions of very low signal intensity can occur even at TE ≈ 0 ms due to severe susceptibility-induced T2* shortening. In such cases, the reduced signal-to-noise ratio can result in inaccurate susceptibility source separation. While this limitation did not affect the results of the current clinical cases in this study, it remains a critical consideration for future studies involving more progressed HA where iron deposition may be more profound.

This study has several limitations. First, this feasibility study included only five patients and evaluated two joint types (knee and ankle), which limits statistical power and generalizability. Susceptibility characteristics and tissue composition vary across joints, and therefore, the findings may not directly translate to other commonly affected joints such as the elbow, shoulder, or hip. As a result, the present findings should be interpreted as a technical proof-of-concept rather than evidence of clinical efficacy. Larger studies including multiple joint types and more comprehensive patient data are needed to validate the clinical applicability of this technique. Second, as noted above, the quantitative accuracy of the estimated paramagnetic and diamagnetic components in hemosiderin-laden tissues has not been validated. This validation can be performed using ground truth obtained from ex vivo tissue analysis and histological studies. We intend to further investigate this in follow-up research.

## 5. Conclusions

This study is the first to show that UTE-based quantitative susceptibility source separation is feasible for MSK imaging. The proposed method improves the detection of paramagnetic substances such as hemosiderin in HA and holds promise for better characterization of bone and joint tissue composition by overcoming susceptibility cancellation effects.

## Data Availability

The data presented in this study are available on request from the corresponding author due to privacy.

## Abbreviations

The following abbreviations are used in this manuscript:

ppm	Parts per million
ppb	Parts per billion
QSM	Quantitative susceptibility mapping
MSK	Musculoskeletal
T2	Spin-spin relaxation time
UTE	Ultrashort echo time
UTE-QSM	Ultrashort echo time-based QSM
HA	Hemophilic arthropathy
IDEAL	Iterative decomposition of water and fat with echo asymmetry and least-squares estimation
T2*	Effective spin-spin relaxation time
PDF	Projection onto dipole field
UTE-DESS	Ultrashort echo time double echo steady state
